# Myoepithelioma-Like Tumor of the Vulva

**DOI:** 10.1155/crip/2100330

**Published:** 2025-01-19

**Authors:** Jiansheng Ma, Qingda Meng, Fangshu Chen, Jiang Wei

**Affiliations:** ^1^Department of Obstetrics and Gynecology, Zhangqiu District Hospital, Jinan City, Shandong Province, China; ^2^Department of Neurointerventional, Zhangqiu District Hospital, Jinan City, Shandong Province, China; ^3^Cardio-Thoracic Surgery, Zhangqiu District Hospital, Jinan City, Shandong Province, China

**Keywords:** INI1/SMARCB1, MELTVR, vulva

## Abstract

**Background:** Myoepithelioma-like tumor of the vulvar region (MELTVR) is a rare mesenchymal tumor that typically arises in the female vulva.

**Case Presentation:** Here, we report a case of a 48-year-old woman who presented with a 2-year history of subcutaneous mass in the vulvar region. As the mass rapidly increased in the last 2 months, personal slight swelling pain appeared. Histologically, the tumor exhibited a distinctive feature of abundant tumor cells and sparse mucus regions. While each region appeared alternately, the sparse mucus region was about 30% of the whole tumor. The tumor had two types of cells, namely, epithelioid and spindle cells. The tumor-rich region demonstrated a cell type of epithelioid, showing hermaphroditic cytoplasm, the center-located nucleus, and abundant chromatin of fasciculate or cord-braid arrangement, whereas the cell of the mucus region was fusiform or epithelioid with partial vacuole-shaped and small visible nucleolus, exhibiting red-stained cytoplasm and loose chromatin. Immunohistochemically, vimentin, smooth muscle actin (SMA), and P16 were diffuse positive in tumor cells, whereas desmin, cytokeratin (CK), P40, P63, CK5, HMB45, MyoD1, myogenin, S100, and SOX10 were all negative. While the proliferation index of Ki-67 was about 7%, the expression of SMARCBl/lNI-1 protein was absent. The pathological diagnosis is myoepithelioma-like tumor of the vulva (right labia majora). Finally, the tumor was surgically and completely removed, and no recurrence or metastasis was found after 6 months of follow-up.

**Conclusions:** Histologically, the morphology of MELTVR is changeable and variation existed for each individual tumor. Moreover, it needs to be differentiated from various other types of tumors, whereas more reports and studies are required to further clarify MELTVR.

## 1. Introduction

Myoepithelioma-like tumor of the vulvar region (MELTVR) is a rare mesenchymal tumor occurring in the female vulva. In 2015, Folpe et al. reported 14 cases of vulvar tumors with INI1/SMARCB1 deficiency, indicating that the vulva is the high-incidence site of this tumor [1]. In the same year, Yoshida et al. reported nine cases of vulvar tumors with special immunohistochemical characteristics, including estrogen receptor positive and S100 protein and glial fibrillary acidic protein negative with simultaneously losing expression of INI1/SMARCB1, and named them MELTVR [2]. Since then, four cases have been reported in the literature [3–6]. This tumor has not been included in the fourth edition of WHO about soft tissue tumor classification [7], easily leading to misdiagnosis. Therefore, more reports or studies are apparently needed to further clarify this type of tumor. Here, we diagnosed one case of MELTVR and the clinicopathological features of the tumor were discussed combining with related literature in order to better understand the tumor (Table 1).

## 2. Data and Methods

### 2.1. Clinical Data

The patient is a 48-year-old woman. Two years ago, she found a tumor on the right labia majora accidentally. At first, the tumor was about the size of a peanut and the patient had no special discomfort. However, the mass rapidly increased in the last 2 months, leading to personal slight swelling pain that caused admittance of the patient into our hospital for a treatment. During gynecological examination, the subcutaneous round mass of the right labia majora is about 3 cm in diameter, soft, with moderate mobility, clear boundary, mild tenderness, and no swelling and ulceration of the surface skin. Color Doppler ultrasound showed subcutaneous cystic solid mass of the labia majora. The clinical diagnosis is subcutaneous lipoma. The tumor was surgically and completely removed, and no recurrence or metastasis was found after 6 months of follow-up.

### 2.2. Methods

Surgical specimens were fixed with 10% neutral formalin, embedded in paraffin, sectioned 4 *μ*m, stained with HE, and observed under a light microscope. Immunohistochemical staining was performed by streptavidin–peroxidase (SP) method. The antibodies desmin, CK, Ki67, P16, P40, P63, smooth muscle actin (SMA), vimentin, CK5, HMB45, INI-1, MyoD1, myogenin, S100, and SOX10 were all purchased from Beijing Zhongshan Jinqiao Biotechnology Co. Ltd., and immunostainings were performed according to the instructions.

## 3. Results

Grossly, a piece of irregular soft tissue with gray–white ribbon skin of a size about 4 cm × 3.5 cm × 3 cm was roughly examined, demonstrating the skin area of about 4 cm × 1 cm and a nodule with a size of about 3 cm × 3 cm × 2 cm under the skin. The section was lobulated, showing grayish yellow between gray and white with medium–soft and clear boundary.

**Figure 1:**
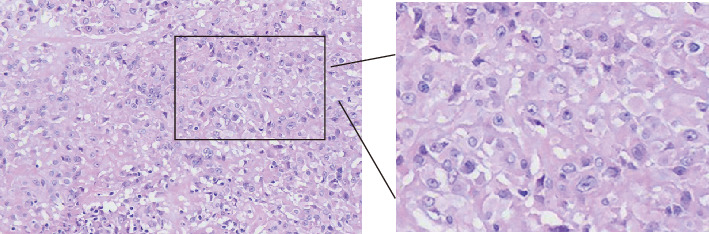
Histological analysis of the growth. Tumor cells resemble epithelial cells; they are loosely packed into sheets or meshes. About 30% of the tumor is made up of sparse mucous areas (H&E stain, 200 × magnification, with the images magnified three times).

Histologically, the tumor was lobulated, exhibiting complete fibrous pseudocapsule and fibrous division without necrosis. The most striking feature of the tumor contained both abundant tumor cells and sparse mucus regions. In contrast, each of the areas appeared alternately and the sparse mucus region was about 30% of the whole tumor. The tumor contained two types of tumor cells, namely, epithelioid and spindle cells. The tumor cell–rich area was epithelioid tumor cells which were amphoteric cytoplasm, center-located nucleus, and abundant chromatin with an arrangement of bundles or strips. The cells in the mucus area are spindle-shaped or epithelioid with partial vacuole-shaped and small visible nucleolus, demonstrating red cytoplasm and sparse chromatin, whereas some cells were mitotic with three to five mitotic images in every 10 high-magnification fields and the nuclei exhibited low grade. The tumor cells were loose and arranged in sheets or meshes (Figure 1). While the interstitial blood vessels of the tumor are rich and branched with about 10% of the area occurring glassy transformation, the tumor was infiltrated by few lymphocytes, plasma cells, and eosinophils (Figure 1).

**Figure 2:**
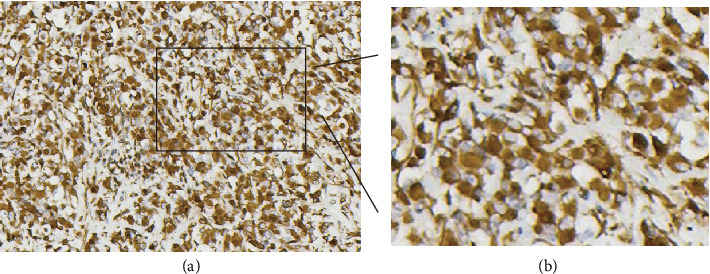
Immunohistochemical staining of vimentin in the tumor. (a) Staining of vimentin (+) by using the EnVision method. (b) Magnification image of the inset. The image was magnified three times.

**Figure 3:**
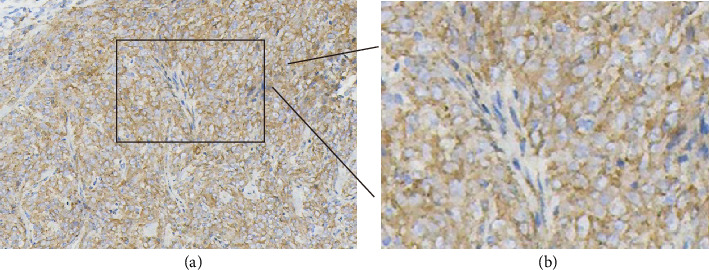
Immunohistochemical staining of the tumor for smooth muscle actin (SMA). (a) Staining of SMA (+) by using the EnVision method. (b) Magnification image of the inset. The image was magnified three times.

**Figure 4:**
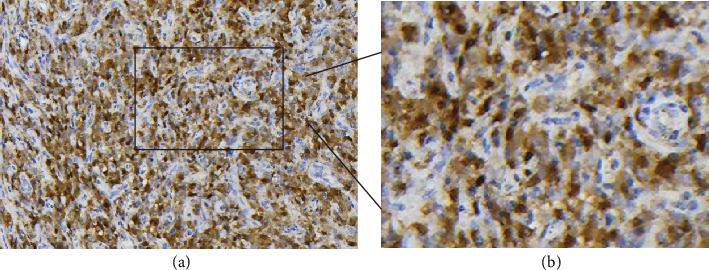
P16 in the tumor stained with immunohistochemistry. (a) Staining of P16 (+) using the EnVision method. (b) Magnification image of the inset. The image was magnified three times.

Immunohistochemically, vimentin (Figure 2), SMA (Figure 3), and P16 (Figure 4) were diffuse positive in tumor cells, whereas desmin, CK, P40, P63, CK5, HMB45, MyoD1, myogenin, S100, and SOX10 were all negative. SMARCBl/lNI-1 protein expression was absent, and the proliferation index of Ki-67 was about 7%. The pathological diagnosis is myoepithelioma-like tumor of the vulva (right labia majora).

## 4. Discussion

MELTVR is a rare mesenchymal tumor without expression of INI1/SMARCB1, usually occurring in adult women. While the onset age is 24–70 years old, the average onset age is 44–59 years old. The tumor occurs in the superficial tissues of the vulva and surrounding areas, whereas the onset site of either the labia majora or pubic mound is the most common one, accounting for 70.59% of the incidence site. In addition, other parts such as the perineum or inguinal area might also be the site of MELTVR. The medical history ranged from 1 month to 8 years with an average of 21.38 months. MELTVR, which was easily misdiagnosed as lipoma, hemangioma, leiomyoma, fibroma, and other tumors before operation, demonstrated a well-defined superficial tumor under the vulva and a few patients were accompanied by pain [4, 5].

MELTVR is a lobulated tumor with clear boundary, complete capsule, grayish yellow, accompanying with or without mucus denaturation. Additionally, most of the tumor is medium–soft or soft with good mobility, whereas hard-textured tumor can occasionally be seen [3]. Microscopically, while the tumor boundary is generally clear with fibrous pseudocapsule and fibrous division, some cases have tiny extracapsular infiltration and the invasive growth boundaries [4]. Their proportions of mucus areas are different and mucus-like areas account for 5%–95% with an average of 54.17%, whereas 35.29% area of the tumors can be necrotic. Consistent to the literature, we observed that the tumor consists of epithelioid and spindle cells. Moreover, we found that the tumor cell–rich area, which were arranged in bundles or strips, was epithelioid tumor cells with amphoteric cytoplasm, center-located nucleus, and abundant chromatin. In contrast, the cells in the mucus area were spindle-shaped or epithelioid with red cytoplasm and sparse chromatin, whereas some cells were vacuolar with small nucleoli. Interestingly, although mitotic images are rare, some cells were mitotic with the low-atypic nuclei and loosely arranged in sheets or meshes. The interstitial blood vessels of the tumor are rich and branched with occurrence of glassy transformation in some areas, whereas a few lymphocytes, plasma cells, and eosinophils infiltrated around the tumor. According to former studies [1–3], MELTVR can also have a morphology of either rhabdoid or similar solitary fibrous tumor. In addition, the tumor is easily misdiagnosed as rhabdomyosarcoma and solitary fibroma in clinic and needs to be differentiated from the aforementioned tumors. Immunohistochemical examination showed that tumor cells were strongly positive for vimentin, SMA, and P16 but negative for desmin, CK, P40, P63, CK5, HMB45, MyoD1, myogenin, S100, and SOX10. Basically consistent with the relevant literature reports, we found that, while expression of SMARCBl/lNI-1 was absent, the proliferation index of Ki-67 was about 7%. In one study [4], it has reported that there is also a case of invasive growth of MELTVR with only containing a few estrogen receptor–positive cells (< 1%) in the noninvasive area, indicating that the variability does exist in the tumor.

MELTVR usually consists of mucus-like areas with epithelioid to spindle-shaped tumor cells, exhibiting similarly as that of soft tissue epithelioma. Recent studies have showed that MELTVRs had internal tissue heterogeneity and demonstrated extensive histological variation. Based on the aforementioned characteristics, it should be differentiated from soft tissue myoepithelioma, epithelioid sarcoma, deep fibrous histiocytoma, and cellular angiofibroma when the diagnosis takes place. MELTVRs and soft tissue myoepithelioma have similar histological features and changes, such as myxoid degeneration, whereas about 10% of soft tissue myoepithelioma, which is the first tumor to be identified, lack immune staining of SMARCB1 [8]. Different from myoepithelioma, the main part of MELTVRs is the nonmucus region positive for both receptors of estrogen and progesterone. In contrast, soft tissue myoepithelioma mainly expresses S100, CK, etc., whereas EWSR1 gene is often ectopic, resulting in EWSR1-POU5F1 gene fusion [9]. Epithelioid sarcoma can occur in the vulva region, occurring with invasive growth, polymorphic nucleus, obvious cell atypia, and vesicular nucleus. Furthermore, most epithelioid sarcomas are positive for CD34 and negative for ER, although there are rare cases of epithelioid sarcoma with myxoid degeneration [10], causing difficulty in identifying them from MELTVR. Since deep fibrous histiocytoma and cellular angiofibroma usually lack mucus-like areas and androgynous cytoplasm, demonstrating epithelial cytological changes of cellular angiofibroma [11, 12], they can also be excluded according to the histological and immunological characteristics of MELTVRs.

Among 17 cases that have been reported, 4 of them had vascular infiltration, whereas 3 cases had regeneration or recurrence after resection of the lesion. In these cases, the margins of resection were all positive for MELTVR and the recurrence obviously was related to surgical factors. In all cases, none of them had lymph node metastasis or distant organ metastasis and the patients did not receive further treatment after operation. In addition, after an average 44 months of follow-up, all of the patients survived.

In summary, the morphology of MELTVR is changeable and variation has existed for each individual tumor. Furthermore, it needs to be differentiated from various other types of tumors and more studies are required to further clarify MELTVR.

## Figures and Tables

**Table 1:** Clinical and pathological manifestations of 13 cases of MELTVR.

**Serial number**	**Author**	**Age (years)**	**Position**	**Tumor size (cm)**	**Time of onset (month)**	**Mucinous area (%)**	**Necrosis**	**Nuclear atypia**	**Mitotic figure (/10 HPF)**	**Vascular infiltration**	**Recrudescence**	**Metastasis**	**Follow-up time (months)**
1	Yoshida	49	Labia majus	4.3	72	5	Yes	Low	6	No	No	No	15
2	Yoshida	42	Labia majus	7.0	> 60	60	No	Low	4	Yes	Yes	No	26
3	Yoshida	24	Labia majus	3.3	1	75	No	High	8	Yes	No	No	87
4	Yoshida	41	Labia majus	2.0	10	95	No	High	2	No	Yes	No	14
5	Yoshida	52	Mons pubis	7.7	92	20	Yes	High	12	Yes	No	No	10
6	Yoshida	28	Labia majus	3.0	5	80	Yes	High	2	No	No	No	101
7	Yoshida	35	Groin	2.4	3	90	No	High	7	No	No	No	172
8	Yoshida	65	Groin	3.2	6	95	No	High	11	No	No	No	1
9	Yoshida	35	Mons pubis	#	12	< 5	No	High	< 1	No	Yes	No	170
10	Kaku	31	Mons pubis	2.0	13	10	No	High	3	No	No	No	11
11	Kojima	70	Groin	3.6	12	40	Yes	High	2–3	Yes	No	No	12
12	Xu	65	Perineum	5.5	72	20	No	Low	1	No	No	No	8
13	Zhang	34	Mons pubis	3.0	6	60	No	High	6	No	No	No	6

*Note:* The field of view # of HPF (high-power field) high-power mirror is described as the size of table tennis.

## Data Availability

All data supporting the findings of this manuscript are readily available.

## References

[1] Folpe A. L., Schoolmeester K., McCluggage W. G. (2015). SMARCB1-deficient vulvar neoplasms. *The American Journal of Surgical Pathology*.

[2] Yoshida A., Yoshida H., Yoshida M. (2015). Myoepithelioma-like tumors of the vulvar region. *The American Journal of Surgical Pathology*.

[3] Kaku Y., Goto K., Kabashima K. (2016). Myoepithelioma-like tumor of the vulvar region presenting as a nonmyxoid spindle-cell neoplasm: a potential histologic mimicker of solitary fibrous tumor. *The American Journal of Dermatopathology*.

[4] Kojima Y., Tanabe M., Kato I. (2019). Myoepithelioma-like tumor of the vulvar region showing infiltrative growth and harboring only a few estrogen receptor-positive cells: a case report. *Pathology International*.

[5] Xu Y., Gao H., Gao J. L. (2020). Myoepithelioma-like tumor of the vulvar region: a case report in China and review of the literature. *Diagnostic Pathology*.

[6] Zhang H. Z., Wang S. Y. (2019). Myoepithelioma-like tumour of the vulvar region. *Pathology*.

[7] Fletcher C., Bridge J. A., Hogendoorn P. C., Mertens F. (2013). *WHO Classification of Tumours of Soft Tissue and Bone: WHO Classification of Tumours, vol. 5*.

[8] Hornick J. L., Dal Cin P., Fletcher C. D. (2009). Loss of INI1 expression is characteristic of both conventional and proximal-type epithelioid sarcoma. *The American Journal of Surgical Pathology*.

[9] Antonescu C. R., Zhang L., Chang N. E. (2010). EWSR1‐POU5F1fusion in soft tissue myoepithelial tumors. A molecular analysis of sixty-six cases, including soft tissue, bone, and visceral lesions, showing common involvement of theEWSR1gene. *Genes, Chromosomes & Cancer*.

[10] Flucke U., Hulsebos T. J., van Krieken J. H., Mentzel T. (2010). Myxoid epithelioid sarcoma: a diagnostic challenge. A report on six cases. *Histopathology*.

[11] Gleason B. C., Fletcher C. D. (2008). Deep “benign” fibrous histiocytoma: clinicopathologic analysis of 69 cases of a rare tumor indicating occasional metastatic potential. *The American Journal of Surgical Pathology*.

[12] Chen E., Fletcher C. D. (2010). Cellular angiofibroma with atypia or sarcomatous transformation: clinicopathologic analysis of 13 cases. *The American Journal of Surgical Pathology*.

